# Effects of Ball Milling Time and Sintering Temperature on the Microstructure and Mechanical Properties of Mg-Al-Ti Alloy

**DOI:** 10.3390/ma18214936

**Published:** 2025-10-29

**Authors:** Dan Qian, Yue Shen, Zhanli Geng, Binyu Zhao, Wandong Bai, Shiping Sun, Xiang Li, Jinbo Zeng, Shengdi Zhang, Yumin Wang, Xiufeng Ren

**Affiliations:** 1 Key Laboratory of Green and High-End Utilization of Salt Lake Resources, Qinghai Institute of Salt Lakes, Chinese Academy of Sciences, Xining 810008, China; qiandan23@mails.ucas.ac.cn (D.Q.); shenyue@isl.ac.cn (Y.S.); gengzhanli@isl.ac.cn (Z.G.); binyuzhao@isl.ac.cn (B.Z.); bwd@isl.ac.cn (W.B.); lixiang@isl.ac.cn (X.L.); jinbozeng@isl.ac.cn (J.Z.); zhangshengdi@isl.ac.cn (S.Z.); wangyumin23@mails.ucas.ac.cn (Y.W.); 2 Qinghai Provincial Key Laboratory of Resources and Chemistry of Salt Lakes, Xining 810008, China; ssp171@163.com; 3 University of Chinese Academy of Sciences, Beijing 100049, China

**Keywords:** Mg, Ti, ball milling, sintering temperature

## Abstract

Driven by the demand for lightweight materials, magnesium has gained significant interest due to its abundance and low density. This study systematically investigated the effects of mechanical ball milling time and sintering temperature on the microstructure and mechanical properties of a powder-metallurgy-processed Mg-Al-Ti alloy. The results established a correlation between ball milling and sintering processes, demonstrating that regulating precursor powder characteristics effectively enhances sintering diffusion efficiency. By precisely controlling sintering temperature and powder particle size characteristics, the alloy achieved high density, hardness, and strength at relatively low temperatures, demonstrating comprehensive performance. Optimal properties were obtained at 420 °C sintering conditions: relative density of 98%, hardness of 172 HV, compressive strength of 367 MPa, and nanoscale Young’s modulus reaching 45.15 GPa. Further analysis indicated that intermetallic compounds formed during sintering contributed significantly to the hardness enhancement, with the strengthening mechanism primarily attributed to the synergistic effects of precipitation and solid solution strengthening. The work provides a theoretical basis for further development of high-performance materials by subsequent processing.

## 1. Introduction

With the advancement of metallic materials science and the growing demand for lightweight structural applications, magnesium alloys and their composites have garnered significant attention [1,2,3]. These materials, known for their cost-effectiveness and natural abundance, are characterized by low density, high specific stiffness, and exceptional specific strength [4,5]. Currently, they are widely used in aerospace, automobile manufacturing, and other engineering fields [6,7]. However, broader applications of magnesium alloys are limited by their inherent drawbacks, including relatively low elastic modulus, inadequate wear resistance, and poor corrosion resistance [8,9]. Notably, the addition of alloying elements or reinforcements has been proven effective in mitigating these drawbacks [10,11]. Considerable efforts have been made to enhance their room-temperature mechanical properties by introducing reinforcements such as SiC, AlN, Y_2_O_3_, etc. [12,13]. Among them, hard metallic Ti particles stand out as the ideal reinforcement due to their high melting point, superior Young’s modulus, and favorable lattice matching with Mg. The lattice compatibility enables Ti particles to significantly improve the strength and wear resistance of magnesium alloys while retaining their lightweight advantages [14,15]. However, the scarcity of reliable thermodynamic data in the Mg-Ti phase diagram, particularly concerning the intermetallic formation temperatures and solubility limits, poses a significant obstacle to the controlled incorporation of Ti particles into the magnesium matrix. In addition, the efficacy of Ti in enhancing the mechanical properties of composites is governed by two critical factors: the homogeneity of titanium dispersion and the adhesion at the titanium-magnesium interface. These factors are highly dependent on the fabrication routes. Consequently, process engineering offers a feasible pathway for optimizing composite performance.

Common methods of preparing magnesium alloys include casting, additive manufacturing and powder metallurgy (PM) [16]. Casting alloys often suffer from reduced performance due to defects such as chemical composition heterogeneity and coarse-grain morphology, while additive manufacturing can produce fine microstructures and improved material properties [17]. However, the complex process and high cost of additive manufacturing restrict its large-scale application [18]. Meanwhile, owing to the substantial density discrepancy between Mg and Ti, the traditional casting method causes Ti to settle rapidly to the bottom, resulting in its inhomogeneous distribution within the alloy. In contrast, the distribution of Ti particles in metal matrix composites fabricated via powder metallurgy (PM) can be relatively uniform. PM—integrating powder consolidation and sintering process—offers dual advantages: (i) produces materials with high density and excellent mechanical properties; (ii) features a simple process and low cost [19]. The sintering process, a critical step in PM, involves two interconnected mechanisms: densification and grain growth. They are strongly influenced by thermal diffusion, including both grain boundary and bulk diffusion, which governs mass transport during the sintering process [20]. Under suitable conditions of powder particle characteristics (size, shape, distribution) and sintering parameters, this diffusion significantly enhances bonding at grain interfaces, thereby influencing the microstructure and final properties of the material [21]. Moreover, ball milling not only enables a more homogeneous powder mixture but also has a remarkable influence on the size and morphology of the powder mixture, through controlling ball-to-powder ratio (BPR), rotational speed, and ball milling time [22]. Numerous studies have explored these factors. For example, Wang et al. [23] systematically investigated the effects of ball milling time, compaction pressure, and sintering temperature on the microstructure and mechanical properties of materials in powder metallurgy. And demonstrated that the relative density and hardness of the samples can be optimized under suitable process conditions. Adhikari et al. [24] revealed that high-energy milling successfully reduced the particle size of AM60 alloy to 22 μm and introduced micropores, resulting in an improvement in total pore volume and hydrogen absorption capacity. Kumar et al. [25] found that the densification of polycrystalline magnesium ferrate (MgFe_2_O_4_) ceramics increased with increasing sintering temperature.

Based on the above discussion, optimizing process conditions—particularly ball milling time and sintering temperature—is essential for effective control of thermal diffusion during sintering and achieving desirable alloy properties. However, research on the preparation and process optimization of Mg-Al-Ti alloys via powder metallurgy remains limited. To address this gap, this work designed two Mg-Al-Ti alloys and systematically investigated the effects of ball milling time and sintering temperature on their microstructure and mechanical properties. The findings establish optimal powder metallurgy parameters and provide a theoretical basis for further performance enhancement by subsequent processing.

## 2. Materials and Methods

### 2.1. Synthesis of Mg-Al-Ti Alloys

**Figure 1:**
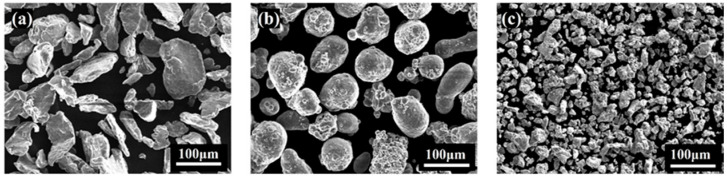
Morphologies of raw material powders: (**a**) Mg; (**b**) Al; (**c**) Ti.

Commercially available magnesium powder (≥99%, 75–150 μm, Aladdin, Shanghai, China), aluminum powder (≥99%, 75–150 μm, Aladdin, Shanghai, China), and titanium powder (≥99.8%, 45–50 μm, Aladdin, Shanghai, China) were utilized. The initial morphologies of raw material powders are presented in Figure 1a–c, respectively.

P1 and P2 were synthesized through a combination of ball milling and conventional sintering, and their specific compositions are detailed in Table 1. The detailed process parameters are provided in Table 2. Based on literature review and our preliminary exploratory experiments (details available in Supporting Materials), the ball milling time range has been narrowed to 80–640 min, and the sintering temperature range has been fixed at 385–445 °C.

We conducted experiments on two groups of samples, P1 and P2, with milling durations ranging from 80 to 640 min. Based on the relative density and hardness measurements of the resulting samples, a comprehensive evaluation indicated that the P2 group exhibited superior performance. Therefore, the P2 samples were selected for further investigation to determine the optimal sintering temperature.

During the ball milling, the powder mixture of the Mg-Al-Ti system was fabricated by using a planetary ball mill (JX-4G made by Shanghai Jingxin, Shanghai, China), which fits with a Ф 10 cm milling jar and Ф 1, 5, 10 mm agate grinding media. The ratio of grinding balls was determined based on literature review and experience as Ф 1:5:10 mm = 2:5:3. And the entire ball milling process was conducted at room temperature. The powders were subjected to milling for different milling times by using a ball-to-powder ratio (BPR) of 3:1 and a rotation speed of 250 rpm. A milling–pause cycle consisting of 10 min of milling followed by a 10 min interval was implemented throughout the entire process to prevent excessive temperature increases within the milling jar. After ball milling, the homogeneously mixed powder was retrieved and compacted into green bodies at a pressure of 35 MPa with a holding time of 10 min. The green bodies were placed in a tube furnace (KSL-1700X, Hefei, China) and sintered under an Ar atmosphere.

### 2.2. Microstructure and Mechanical Properties Characterization

All microstructural characterization techniques and performance tests involved in this work were conducted at room temperature. X-ray diffractometer (XRD; D8 Discover, Karlsruhe, Germany) was used to examine the phase composition of the samples with the following parameters: Cu target (Kα1, wavelength 0.15406 nm), 40 kV, 40 mA, scanning step 0.02°, and slow scanning step 0.01°. A scanning electron microscope (SEM, HITACHI SU8010, Tokyo, Japan) was employed to characterize the size and morphology of the powder mixture. The surface micromorphology of sintered samples was examined by SEM and a 3D Scandisk confocal microscope (MahrSurf CM explorer, Mahr, Gottingen, Germany). The particle size and distribution of the powder mixture were examined by laser particle sizer (Mastersizer 3000, Malvern, UK), while SEM images were statistically analyzed for particle size by Image J (1.8.0.345) software. Prior to testing, all sintered samples were ground with sandpaper of varying grit sizes (800, 1000, 1200, 1500, and 2000) and subsequently polished with diamond polishing paste.

Theoretical density was calculated using the rule of mixtures (Equation (1)), while the actual density of the sintered samples was determined using Archimedes’ principle. The relative density was calculated according to Equations (1) and (2) in order to evaluate the extent of sample densification.

(1) $$\rho_{t}=\Sigma_{\mathrm{i}=1}^{n}\left(V_{1}\rho_{i}\right)$$

where $V_{1}$, $\rho_{i}$ represent the volume fraction and density of phase i, respectively.

(2) $$\rho_{r}=\rho_{s}/\rho_{t}$$

where $\rho_{r}$, $\rho_{s}$, and $\rho_{t}$ represent relative density, actual density, and theoretical density, respectively.

An atomic force microscope (AFM, Dimension Icon, Bruker, Billerica, MA, USA) with the Peak Force quantitative nanomechanical mapping (PF-QNM) mode was employed to obtain the morphology and mechanical properties simultaneously. The probe used was DNISP-HS (350 N/m, 50 kHz, Bruker), and its applicable sample modulus range was 10–100 GPa, while its tip radius was estimated by the relative method using a standard sample (fused silica). The indentation depth was relatively dependent on the peak force set point and controlled between 2.0–10.0 nm to ensure the reliability of the modulus data. Microhardness was conducted through a Vickers hardness tester (MHVS-50T, Shanghai, China), which measured five areas consecutively to obtain an average value. The quasi-static compressive mechanical property of sintered samples was measured using a microcomputer electronic universal testing machine (CMT5305, Ningbo, China) under a strain rate of 1 mm∙min^−1^.

## 3. Results

### 3.1. Effect of Ball Milling Time

As a fundamental process in the powder metallurgy of magnesium alloys, ball milling directly determines the quality of the precursor powder and the resultant properties of the sintered product. The process achieves particle refinement, homogenization, and solid-state alloying via mechanical force, with its efficacy being governed by parameters such as milling time. The parameter critically influences the powder’s attributes, including its particle size distribution, morphological features, induced lattice distortion, and elemental uniformity. It is well established that these attributes are fundamental to the densification and microstructural development during sintering. Therefore, drawing from preliminary experiments and existing literature, a ball milling time range of 80 to 640 min was identified as the focus for this study.

#### 3.1.1. Particle Size and Distribution of Ball-Milled Powders

**Figure 2:**
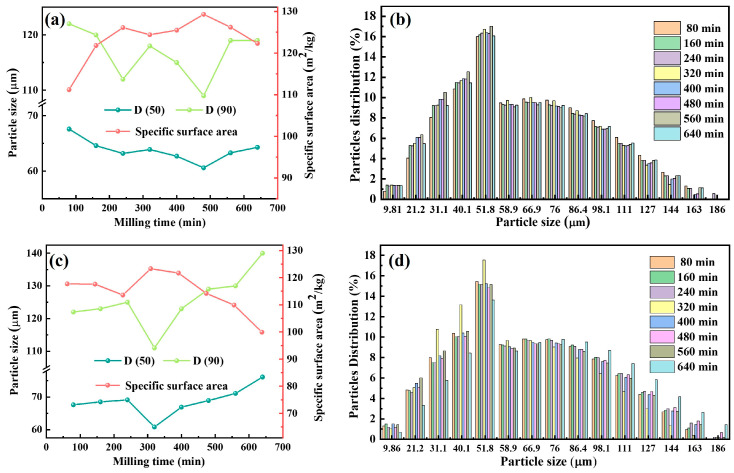
Particle size and its distribution of ball-milled powders at varied ball milling times: (**a**,**b**) P1; (**c**,**d**) P2.

The particle size and distribution of the raw material powders are key factors influencing the sintering process. Finer particles exhibit larger specific surface areas and higher surface energy, which enhances the thermodynamic driving force and promotes atomic or ionic diffusion through increased contact area and reduced diffusion paths. Laser particle size analysis was performed on powder mixtures subjected to various ball milling times. As illustrated in Figure 2, both P1 and P2 samples exhibited a trend of particle size decreasing followed by an increase with extended ball milling time. For P1, the median particle sizes D (50) and D (90) reached minima of 60.9 μm and 109 μm at 480 min, corresponding to a maximum specific surface area of 129.3 m^2^/kg. Similarly, P2 reached D (50) and D (90) minima of 60.8 μm and 111 μm at 320 min, with a surface area peak at 123.3 m^2^/kg.

During ball milling, the evolution of particle size is governed by the competition between fragmentation and cold welding, both induced by repeated mechanical collisions. These two mechanisms occur simultaneously throughout the process, with the powder particle size decreasing when fragmentation dominates, and conversely increasing when cold welding dominates. In the initial period of the ball milling, the constant collisions of the powder-ball and the powder-jar induce severe plastic deformation and leading to the dislocation proliferation and plugging of the powder. As a result, the fragmentation of the particles begins and generates a large quantity of fine particles. At this stage, fragmentation dominates over cold welding, leading to a reduction in particle size. Subsequently, with continued milling, increased input energy shifts the balance toward cold welding, causing particle size to rise slightly. Moreover, the volume fraction of fine particles generated from the initial period of fragmentation continues to increase, resulting in a greater tendency to agglomerate at this stage [26].

Analysis of the particle size histogram (Figure 2b,d) reveals that the particle size distributions are predominantly within the range of 31.1–111 μm. As previously discussed, fragmentation predominated during the initial stage of ball milling, resulting in a gradual increase in fine particles (≤58.9 μm). In contrast, cold welding became dominant in the later stages, accompanied by an increase in larger particles (≥86.4 μm). Furthermore, comparing P1 and P2, it is found that the time required to reach the minimum particle size is influenced by the sample composition. A reasonable reason may be attributed to the fact that the particle size reduction during ball milling mainly relies on magnesium and titanium particle fragmentation. When the content of magnesium powder in P2 decreases while the content of aluminum increases, it promotes the fragmentation of magnesium particles. Thus, the energy generated by the collision in the initial period of ball milling is more focused on fragmentation, leading to a reduction in the time required for powder refinement [27].

**Figure 3:**
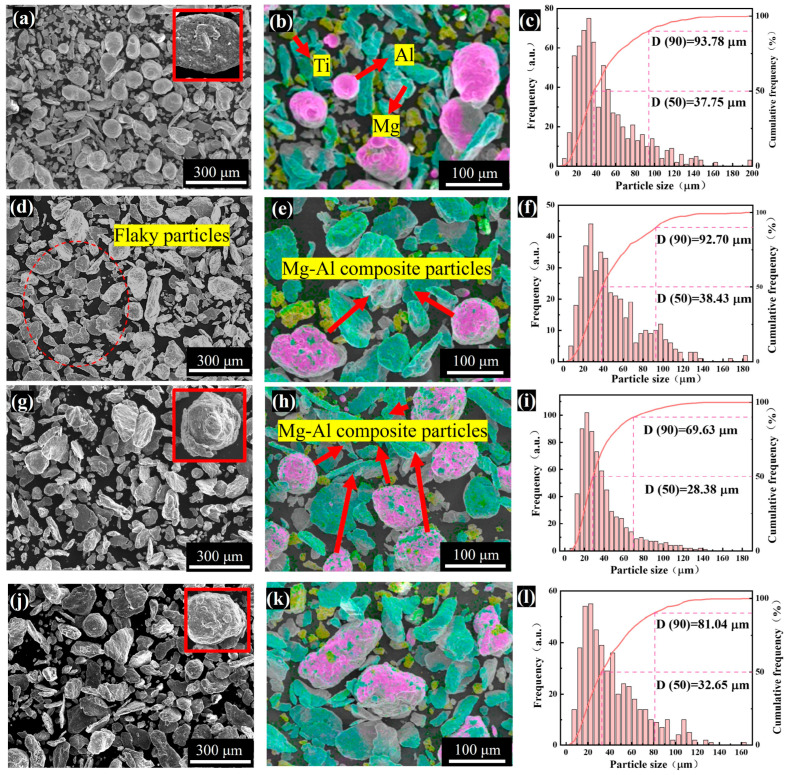
SEM, EDS and particle size statistics of ball-milled powders of P2: (**a**–**c**) 80 min; (**d**–**f**) 240 min; (**g**–**i**) 320 min; (**j**–**l**) 400 min.

As discussed above, the predominant physical process governing powder mixture during ball milling is the synergistic interaction between fragmentation and cold welding. The collision-induced response of the powder system largely depends on the phase composition and microstructural characteristics. More specifically, during the continuous collision process, particles with different crystal structures, hardness and ductility will exhibit varied plastic deformation behavior and interfacial metallurgical bonding characteristics. This mechanism, based on the intrinsic properties of particles, is the fundamental basis for revealing the evolution of the microstructure during the ball milling process. To further elucidate this evolution, SEM and EDS were utilized to observe the morphology of the powders at various milling durations (Figure 3 and Figures S1 and S2).

Both sample groups exhibited comparable trends. In the initial ball milling stage (80 min), the samples primarily consisted of discrete Mg, Al, and Ti particles. Most of these particles retained their original morphology, showing no significant deformation. However, some fine particles were observed adhering to the larger Mg and Al particles (As shown in the inset at the top right of Figure 3a). As milling progressed, brittle Ti particles fragmented into irregular shapes, while ductile Mg and Al particles underwent plastic deformation. Additionally, some Mg particles transitioned from lath-like to flake-like morphologies, with fatigue cracks forming due to accumulated internal stresses (highlighted in Figure S1d). During this period, the dominant mechanism is the plastic deformation mechanism, and the proportion of crushing events is relatively low. Microscopically, cold welding and stress accumulation at particle interfaces became increasingly pronounced. The Mg particles are mainly present as flakes when the ball milling time increases to 240 min (Figure 3d), at which point magnesium-aluminum composite particles begin to appear (Figure 3e) and further increase at 320 min due to enhanced diffusion between magnesium-aluminum particles (Figure 3h). At the same time, the magnesium particles transformed into flakes readily adhere to the surface of aluminum particles, thereby promoting diffusion (As shown in the inset at the top right of Figure 3g,j). Moreover, more energy accumulated by collisions at 320 min leads to stress concentration inside the ductile particles and thus particles fracture, so the number of small-sized particles increases substantially (Figure 3i). Column distribution (Figure 3c,f,i,) analysis confirmed a gradual shift toward smaller particle sizes, with D (50) minima observed at 320 min for P2 and 480 min for P1, consistent with laser particle size measurements. In addition, it is shown that throughout the ball milling process, aluminum is always dominated by the plastic deformation mechanism, and its particle morphology gradually evolves from the initial near-spherical shape to a fibrous ductile structure.

#### 3.1.2. Phase Composition of Ball-Milled Powders

**Figure 4:**
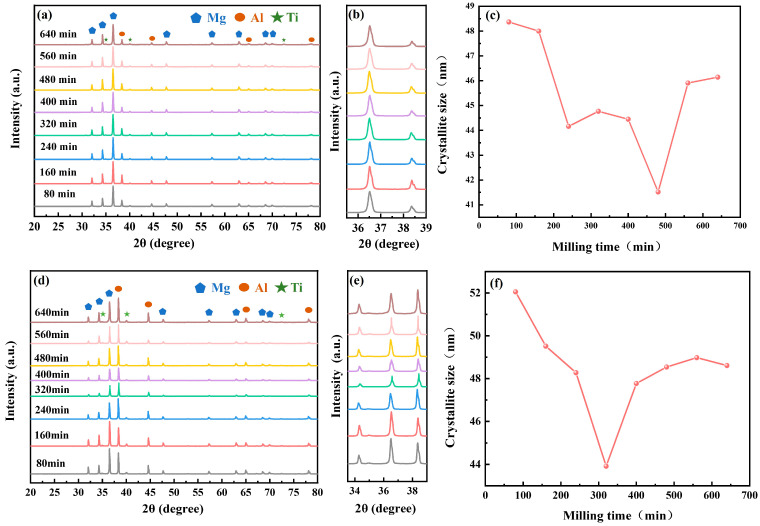
XRD results of ball-milled powders at room temperature and microcrystalline size variation curves with ball milling time: (**a**–**c**) P1; (**d**–**f**) P2.

To further determine whether alloying occurred during the ball milling process, we analyzed the powders at different milling durations using XRD. The results are shown in Figure 4. Diffraction peaks derived from Mg, Al, and Ti can be identified, while no large amounts of other phases were found for all the milling times. Quantitative analysis confirmed that the ratio of the three was basically the same as that of the addition, indicating that the ball milling process was mainly dominated by physical mixing, and no alloying reaction or other chemical reactions occurred. Notably, compared to P1, P2 exhibits a higher aluminum content, resulting in the increase in peak intensity for aluminum in the P2 sample spectrum (Figure 4a,d).

In addition to identifying the physical phases of the powder particles, we further analyzed the powder’s microcrystalline size, lattice strain, and dislocation density using XRD data. After fitting the XRD data by the Lorentz function, it is found that the full width at half peak (FWHM) of the diffraction peaks of the magnesium phase appeared to increase first and then decrease (the specific figure shown in the Supporting Materials). This phenomenon can be attributed to grain refinement caused by plastic deformation and fragmentation, which broadens the diffraction peaks. Whereas the relationship between the microcrystalline size (D), lattice strain (ε), dislocation density (δ) and the full width of half peaks can be mathematically expressed by Stokes-Wilson and Scherrer formulas [28,29,30]:

(3) D=Kλ/cosθ

(4) ε=β/4tanθ

(5) $$\delta =1/D^{2}$$

In the above equation, K is the Scherrer constant, and β, λ, ε, D, and θ represent the full width at half-maximum (FWHM), the wavelength of the incident X-rays, the lattice strain, the microcrystalline size, and the Bragg diffraction angle, respectively. In addition, β subtracts the effect of the instrumental broadening. The data on grain size, lattice distortion, and dislocation density involved in our work were all calculated using Equations (3)–(5).

Figure 4c,f illustrate the evolution of microcrystalline size in P1 and P2 as a function of ball milling time, calculated using Equation (3), respectively. Both samples exhibit a similar trend: the microcrystalline size of Mg is first refined and then coarsened during the ball milling process. Specifically, the microcrystalline size of P1 reached a minimum of 41.52 nm at the milling time of 480 min, and P2 reached a minimum value of 43.92 nm at 320 min. Then the microcrystalline size increased with the prolongation of the ball milling time. Therefore, it can be hypothesized that the microcrystalline size of the sintered samples is positively correlated with the particle size of the ball-milled powder. The reduction in microcrystalline size in the initial period of ball milling essentially originates from the severe plastic deformation induced by continuous collisions. The process drives the grain refinement by continuously increasing the dislocation density and lattice distortion. Specifically, when the dislocation density accumulates to a critical threshold, coarse grains are subdivided into nanoscale subgrains through the multiplication and migration of small-angle grain boundaries, leading to refinement [24]. As the size approaches the critical limit (~100 nm), further plastic deformation becomes increasingly difficult due to enhanced dislocation resistance. At this stage, the dislocation formation mechanism is suppressed, and the lattice strain is attenuated due to grain refinement [31]. Moreover, in the subsequent periods of ball milling, the cold welding process promotes grain agglomeration and localized heating, both of which contribute to grain coarsening [32]. Concurrently, the rearrangement and annihilation mechanism of dislocations will lead to the gradual reduction in dislocation density [33], which explains the evolution of lattice strain and dislocation density with ball milling time (Tables S1 and S2). This is in good agreement with the previous studies [27,28,34].

#### 3.1.3. Phase Composition and Microstructure of Sintered Samples

**Figure 5:**
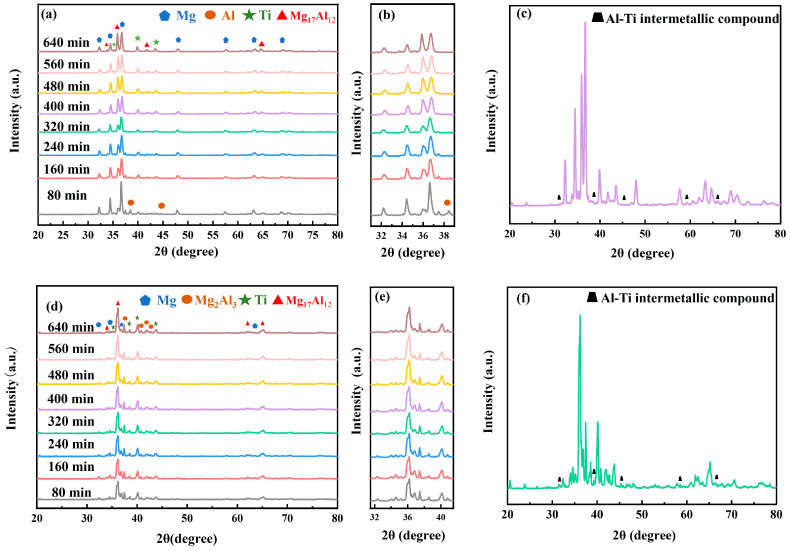
Room-temperature XRD results of sintered samples and slow-scan results: (**a**–**c**) P1; (**d**–**f**) P2.

Given that the ball milling process directly governs powder characteristics—which are, in turn, critical for successful compaction and sintering—optimizing its parameters is essential. Therefore, this study aims to not only systematically analyze the milled powders but also thoroughly evaluate the properties of the sintered samples. This two-pronged approach will provide a comprehensive basis for identifying the optimal processing conditions. Hence, the sintered samples with different ball milling times were analyzed using X-ray diffraction (XRD), and the results are presented in Figure 5.

According to the XRD patterns, it is evident that the sintered samples are mainly composed of Mg, Al, Ti and intermetallic compounds. For P1, the sintered sample milled for 80 min exhibits strong diffraction peaks corresponding to Mg, along with weaker signals from Al, Ti, and Mg_17_Al_12_. Additionally, the residual Al signal indicates incomplete diffusion at this period. With the prolongation of ball milling time, the diffraction peaks of the Al in the sintered sample gradually weakened and disappeared completely after 240 min, accompanied by a progressive intensification of the Mg_17_Al_12_ peaks (Figure 5b). This suggests that Al is fully consumed through dissolution or reaction to form intermetallic compounds. Simultaneously, the Mg peak intensity decreases while the Mg_17_Al_12_ peak becomes more pronounced, indicating that prolonged ball milling time enhances the chemical activity of Mg and Al. This is attributed to the finer powder particles with larger specific surface area, and the accumulation of lattice defects and distortions, which collectively promote atomic diffusion and facilitate the formation of Mg_17_Al_12_ during sintering. For P2, due to the significant enhancement of the aluminum content, the sintering samples are dominated by the Mg_17_Al_12_ phase, accompanied by the generation of the Mg_2_Al_3_ intermediate phase (Figure 5e). The synergistic precipitation of these two reinforcing phases, through the diffuse strengthening mechanism and solid solution strengthening effect, jointly contributes to a significant increase in the hardness of the material. Although the diffraction peak of Ti is very weak, it always exists, and the diffraction peak of Al-Ti intermetallic compounds can be seen under the slow scanning condition (Figure 5e,f).

**Figure 6:**
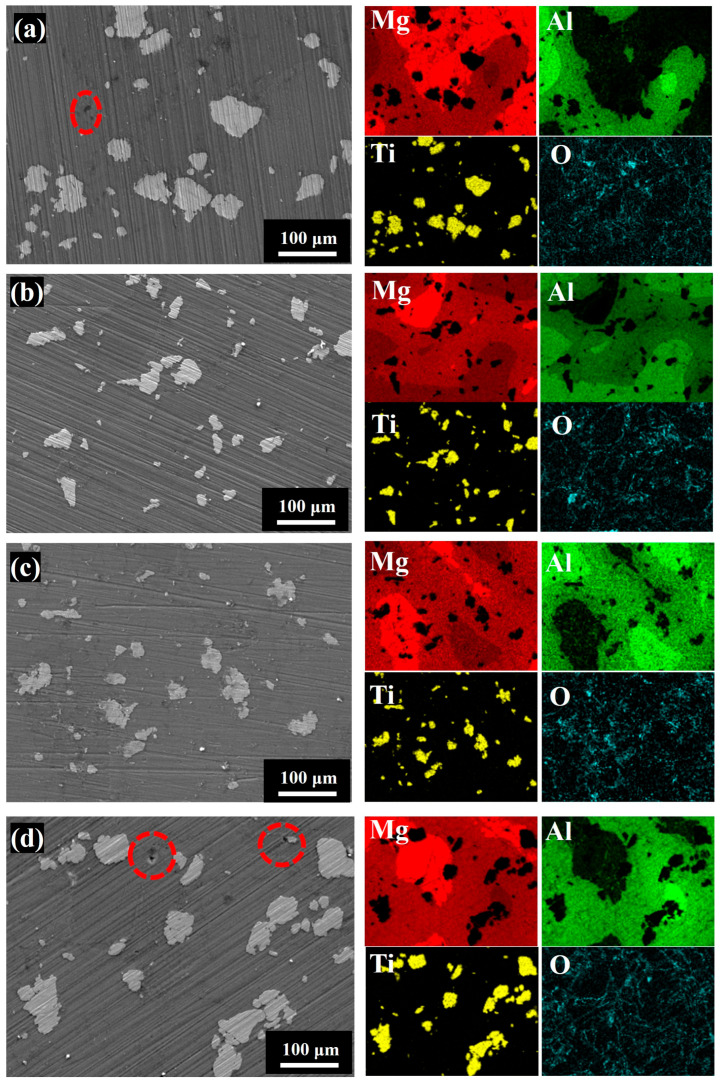
SEM and EDS results of sintered P2 at room temperature: (**a**) 80 min; (**b**) 240 min; (**c**) 320 min; (**d**) 640 min.

Figure S3 and Figure 6 present the SEM images of sintered samples at different ball milling times for P1 and P2, respectively. Owing to the higher aluminum content in P2, the microstructure is predominantly composed of Mg–Al intermetallic compounds, consistent with the XRD results. It is evident that there are large pores in the sintered samples under ball milling for 80 min (Figure S3a), and with the prolongation of the ball milling time up to 480 min, the pores in the microstructure show a decreasing trend. But after 640 min, the sintered body starts to show large pores again (Figure S3d). This trend is closely related to the morphology and mechanical state of the particles. In the initial period of ball milling, the generation of finer particles with high specific surface area improves packing density and promotes sintering, thereby reducing the formation of pores. The prolongation of the ball milling time increases the particle size, and the work-hardening effect of the particles results in an increase in the dislocation slip resistance. These effects hinder atomic diffusion and plastic deformation during sintering, which leads to the formation of pores again. EDS analysis further confirms that aluminum diffusion becomes increasingly pronounced with extended ball milling, promoting the formation of Mg–Al intermetallic phases during sintering. This observation correlates well with the increasing intensity of Mg_17_Al_12_ and Mg_2_Al_3_ peaks in the XRD patterns. In contrast, titanium diffusion remains limited, indicating that Ti primarily exists as a discrete phase with minimal chemical interaction.

#### 3.1.4. Densification and Mechanical Properties of Sintered Samples for Different Milling Times

**Figure 7:**
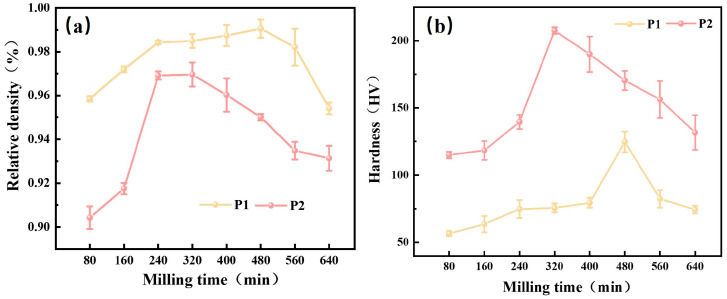
Room-temperature mechanical properties of sintered samples at 420 °C with different ball Milling Times: (**a**) relative density; (**b**) hardness.

To assess the quality of sintered samples under various ball milling times, the theoretical densities were calculated, and the actual densities of the sintered samples were measured (Table S3). The relative densities and hardness of the sintered samples values as a function of milling time are presented in Figure 7.

The relative density of both groups of samples remained at a high level, indicating a high degree of densification. The peak relative densities of 99.06% (for P1 at 480 min) and 97.48% (for P2 at 320 min) were attained, respectively, indicating the optimal ball milling time for densification. The relative density of the sample reaches its peak at the optimal ball milling time, primarily attributed to the fact that under appropriate milling duration, both particle size and energy are maintained at optimal levels. This consequently promotes both diffusion behavior and neck growth during the sintering process, thereby enhancing the sample’s degree of densification [35]. However, with further prolongation of the ball milling time, the increasing brittleness and work-hardening of powder particles reduce their compressibility during pressing. This impairs particle rearrangement and plastic deformation during sintering, leading to reduced densification. Comparative analysis shows that the relative density of P2 remains consistently lower than that of P1. This discrepancy is primarily attributed to the morphological mismatch between the flaky magnesium particles and the near-spherical aluminum particles. The increased Al content exacerbates packing inefficiencies, leading to more irregular pores and thus lowering the overall density.

Figure 7b illustrates the evolution of microhardness with milling time. P1 and P2 reached maximum hardness values of 93.7 HV and 183 HV at 480 and 320 min, respectively. Notably, P2 exhibited both higher hardness and greater variability. This can be mainly attributed to two factors: (1) Densification degree: Fewer pores and improved interparticle bonding with optimal milling enhance bulk hardness. (2) Phase composition: The progressive formation of hard Mg_17_Al_12_ intermetallic compounds contributes to hardness improvement via a diffusion strengthening mechanism. Moreover, the latter contributes more to the hardness. Based on a comprehensive consideration of particle characteristics and final performance, the optimal ball milling times were determined to be P1 (480 min) and P2 (320 min).

### 3.2. Effect of Sintering Parameters

Beyond milling, sintering serves as another critical step governing the final material performance. This process is fundamentally a thermally activated diffusion phenomenon, which drives mass transport to eliminate porosity and achieve microstructural densification.

#### 3.2.1. Microscopic Morphology and Phase Composition of Sintered Samples

**Figure 8:**
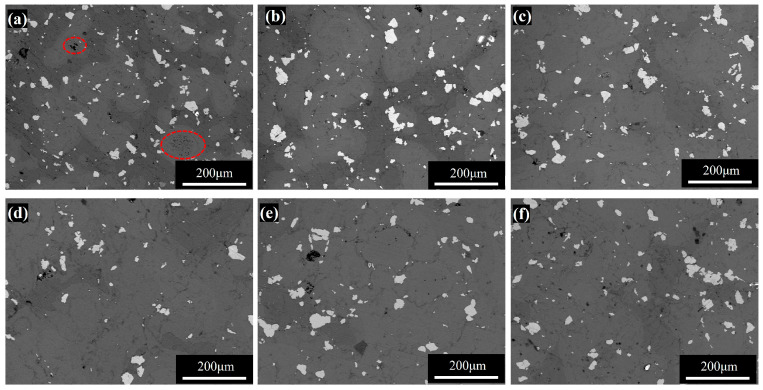
SEM images of P2 sintered at different temperatures: (**a**) 385 °C; (**b**) 405 °C; (**c**) 415 °C; (**d**) 420 °C; (**e**) 425 °C; (**f**) 430 °C.

The sintering temperature and diffusion rate can be expressed by the Arrhenius equation D = D_0_·exp(−Q/RT), where D is the diffusion coefficient, which is the core parameter characterizing the diffusion rate; D_0_, Q, R, and T are the frequency factor, diffusion activation energy, gas constant, and absolute temperature, respectively. It can be noticed that the sintering temperature plays a critical role in determining the thermal diffusion rate of constituent elements. For the purpose of sintering optimization, sample P2 (milled for 320 min) was identified as the key candidate due to its superior properties. The sintering temperature range of 385–430 °C was then designated for detailed study. Figure 8 presents the SEM images of the sample surface insulated for 1 h at different sintering temperatures.

In these images, dark gray regions correspond to the magnesium phase, light gray to Mg-Al intermetallic compounds, white to the Ti phase, and black areas indicate pores formed during sintering (enlarged image shown in Supporting Information Figure S4). It can be observed that pores exist at each sintering temperature, and a large number of small and dense pores are distributed in the sintered samples at 385 °C (Figure 8a). As the sintering temperature increases up to 420 °C, these small and dense pores gradually disappear, although isolated pores remain at grain boundaries (Figure 8b–d). This phenomenon is mainly attributed to the fact that the diffusion rate of the elements increases significantly with rising sintering temperature. This promotes interparticle bonding, facilitates pore shrinkage and closure, and contributes to a denser microstructure. Simultaneously, the volume fraction of Mg-Al intermetallic compounds increases with sintering temperature, further confirming that elevated temperatures enhance interdiffusion between Mg and Al, thereby accelerating the formation of intermetallic phases. However, when temperatures are beyond 420 °C, a resurgence in pore formation is observed (Figure 8e,f). This is attributed to the accelerated movement of grain boundaries outpacing pore migration, leading to the detachment of pores from grain boundaries and the subsequent reformation of fine pores.

**Figure 9:**
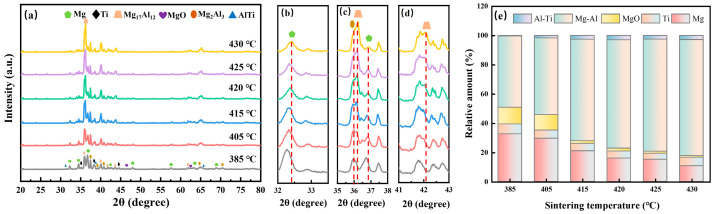
Different sintering temperatures of P2: (**a**–**d**) XRD results at room temperature; (**e**) Relative content of each component.

Figure 9 shows the corresponding XRD results of the P2 samples at various sintering temperatures.

The sintered samples at different temperatures primarily consist of Mg, Ti, oxides, Mg-Al intermetallic compounds (Mg_17_Al_12_, Mg_2_Al_3_), and Al-Ti intermetallic compounds (AlTi, Al_3_Ti, AlTi_3_). The samples sintered at 385 °C showed insufficient sintering characteristics, and the composition was mainly composed of Mg, Ti, MgO, and Mg-Al intermetallic compounds (Mg_17_Al_12_, Mg_2_Al_3_) produced during the sintering process. As the sintering temperature increases, the diffraction peak intensities of Mg and MgO gradually decrease, whereas those of Mg-Al intermetallic compounds show a progressive increase (Figure 9c,d). Notably, a slight shift in the Mg diffraction peaks toward higher angles is observed (Figure 9b,c), indicating the solid solution of a small amount of Al in the Mg lattice. This peak shift results from lattice contraction due to substitutional incorporation of smaller Al atoms (atomic radius: 1.43 Å) into the hcp Mg structure (atomic radius: 1.60 Å). Concurrently, the relative content of Mg and MgO decreases stepwise with rising sintering temperature, while the fraction of Mg-Al intermetallic compounds increases, eventually reaching approximately 80% of the total phase composition. Microstructural analysis further reveals that elevated temperatures promote particle deformation and disrupt oxide layers. This reduction in porosity enables effective metallurgical bonding between particles, ultimately enhancing the alloy’s strength. However, we found remelting overflow on the surface of the alloy at 465 °C, which will cause its performance to be reduced. Additionally, Ti-Al intermetallic compounds (such as AlTi, Al_3_Ti, and AlTi_3_) begin to precipitate and gradually increase in content with rising sintering temperature. These observations collectively demonstrate the strong influence of sintering temperature on atomic diffusion kinetics. Enhanced diffusion at elevated temperatures facilitates the co-precipitation of multiple intermetallic compounds, significantly impacting the microstructural evolution and final properties of the alloy.

#### 3.2.2. Densification and Mechanical Properties of Sintered Samples

**Figure 10:**
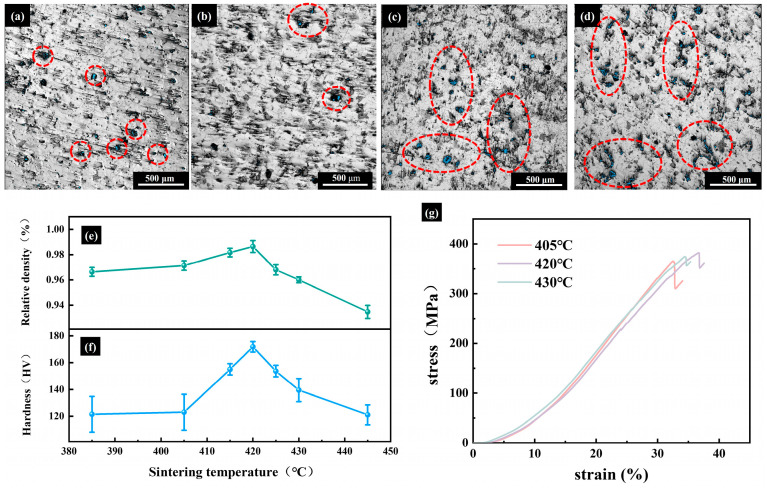
Samples at different sintering temperatures for 320 min. (**a**–**d**) Optical images: (**a**) 415 °C, (**b**) 420 °C, (**c**) 425 °C, (**d**) 430 °C; (**e**) Room-temperature Relative density; (**f**) Room-temperature hardness; (**g**) Room-temperature Compressive stress–strain curves.

Meanwhile, to further investigate the mechanism underlying the hardness evolution of the sintered samples, the surface morphology of P2 samples sintered at different temperatures was systematically analyzed. Figure 10 presents the optical surface images, relative density, hardness variations, and the compressive stress–strain curves of P2 samples.

The specific values for the compression test are listed in Table S4. As shown in Figure 10a–d, the number of dispersed surface pores (marked by dashed circles or ellipses) initially decreases and then increases with rising sintering temperature. This trend aligns well with the simultaneous evolution of the relative density (Figure 10e), which reaches a peak of 98% at 420 °C, indicating a highly densified microstructure conducive to superior mechanical properties. This non-monotonic trend in densification can be understood as follows: during the initial sintering period, elevated temperatures significantly enhance atomic diffusion, effectively promoting pore healing and defect elimination. Concurrently, at moderately high temperatures (below the threshold for abnormal grain growth), normal grain growth contributes to pore filling, resulting in synergistically improved densification. Once the sintering temperature surpasses a critical point, abnormal grain coarsening outpaces pore closure. This leads to the decoupling of grain boundary migration and pore movement, which triggers the re-emergence and expansion of pores. Consequently, when the temperature reaches 430 °C, material densification decreases. This is characterized by a significant increase in pore number, with pores tending to aggregate at grain boundaries. As a result, the relative density drops markedly to 93%.

This behavior closely corresponds to the evolution of material hardness and compression properties (Figure 10f,g). At 385 °C, incomplete densification leads to residual pores and structural defects, which significantly compromise the material’s integrity and lead to a substantial reduction in mechanical performance [36]. Subsequently, the mechanical properties of P2 exhibit a non-linear trend, with the hardness reaching a maximum of 172 HV, and the compressive strength is 382.19 MPa at 420 °C. This variation stems from the combined influence of several factors: during the early temperature rise, the decreasing porosity and increasing fraction of the high-hardness Mg_17_Al_2_ phase both contribute to the enhanced hardness. However, further temperature elevation causes abnormal grain growth, weakening grain boundary strength, and reducing overall densification, which ultimately deteriorates the mechanical performance.

**Figure 11:**
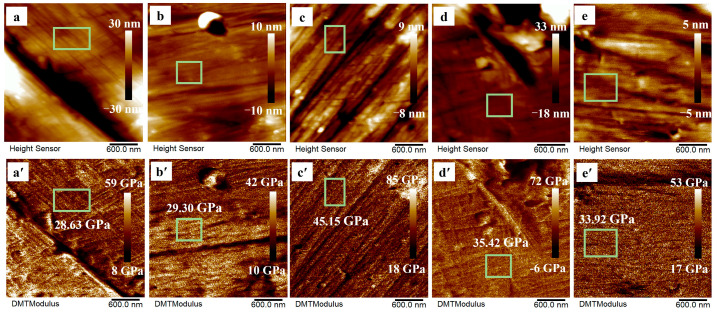
Morphology and nanomechanical properties of P2 at different sintering temperatures for 320 min. (**a**–**e**) Topography height and (**a′**–**e′**) the corresponding DMT modulus images: (**a**,**a′**) 405 °C; (**b**,**b′**) 415 °C; (**c**,**c′**) 420 °C; (**d**,**d′**) 425 °C; (**e**,**e′**) 430 °C.

We further characterized the nanoscale mechanical behavior of P2 samples at different sintering temperatures using AFM in PF-QNM mode. Figure 11a–e′ present the simultaneously captured images of nanoscale morphology and DMT modulus, with the corresponding indentation maps provided in Figure S3. The results indicate that the modulus of P2 initially increases with increasing sintering temperature, peaking at 45.15 GPa at 420 °C. And then decreases at higher temperatures, consistent with macroscopic measurements (Figure 10g). By integrating the concurrent changes in relative density, microstructural development, and mechanical performance, it can be concluded that 420 °C represents the optimal sintering temperature for P2 samples, achieving an effective balance between high densification and superior mechanical strength.

## 4. Conclusions

In this study, we systematically investigated the effects of ball milling time and sintering temperature on the microstructure and mechanical properties of Mg-Al-Ti alloys fabricated via powder metallurgy.

Experimental results indicated that the ball milling process was dominated by physical mixing. Over the milling time range of 80–640 min, the particle characteristics of the milled powders exhibited clear trends. Specifically, the particle size of the P1 powder reached a minimum at 480 min of milling, whereas the P2 powder attained its minimum size at 320 min. Meanwhile, an investigation into the sintering temperature revealed that an optimal temperature simultaneously disrupts oxide layers and enhances atomic interdiffusion. Samples sintered at 420 °C demonstrated the best densification behavior, which is attributed to the synergistic effects of diffusion strengthening and solid solution strengthening. Optimal properties were obtained at 420 °C sintering conditions: relative density of 98%, hardness of 172 HV, compressive strength of 367 MPa, and nanoscale Young’s modulus reaching 45.15 GPa.

The experimental findings confirm that the characteristics of the powder particles and the sintering temperature directly govern the final properties of the sintered samples. And systematically unveils the underlying mechanism of the ball-milling process and establishes a definitive structure-property relationship linking powder characteristics to densification behavior. Leveraging this insight, we have successfully fabricated high-performance magnesium alloys with markedly superior hardness. Our work not only demonstrates a notable advancement in material performance but also paves the way for a novel paradigm in powder metallurgy: the design of sintered materials with predictable and tailorable properties through the targeted engineering of initial powder attributes. These results establish clear processing windows for optimizing Mg-Al-Ti alloys via powder metallurgy and provide a foundation for further enhancement through thermomechanical processing.

## Figures and Tables

**Table 1:** Design composition ratios.

Alloy	Mg (wt%)	Al (wt%)	Ti (wt%)
P1	75	20	5
P2	50	40	10

**Table 2:** The specific parameters for milling and sintering.

Milling Time (min)	Sintering Temperature (°C)	Sintering Time (h)	Heating Rate (°C/min)
80 160 240 320 400 480 560 640	385 405 415 420 425 430 445	1	4

## Data Availability

The original contributions presented in this study are included in the article. Further inquiries can be directed to the corresponding author.

## Supplementary Materials

The following supporting information can be downloaded at https://www.mdpi.com/article/10.3390/ma18214936/s1. Figure S1: SEM, EDS and particle size statistics of P1 with different milling time: (a–c) 80 min; (d–f) 240 min; (g–i) 320 min; (j–l) 400 min; (m–o) 480 min; (p–r) 640 min; Figure S2: SEM, EDS and particle size statistics of P2 with different milling time: (a–c) 480min; (d–f) 640 min; Figure S3: SEM and EDS results of sintered samples of P1 with different ball milling times: (a) 80 min; (b) 240 min; (c) 480 min; (d) 640 min; Figure S4: Indentation maps of P2 at different sintering temperatures: (a) 405 °C; (b) 415 °C; (c) 420 °C; (d) 425 °C; (e) 430 °C; Figure S5: SEM of the sintered sample at 385 °C; Figure S6: Macroscopic morphology of samples prepared at different milling times; Figure S7: Particle size of ball-milled powders at varied ball milling times; Table S1: Lattice strain and dislocation density of P1 ball-milled powders; Table S2: Lattice strain and dislocation density of P2 ball-milled powders; Table S3: Theoretical and actual densities of P1 and P2; Table S4: The specific values of compressive strength, compressive elastic modulus, and fracturestrain of the P2 alloys sintered at different temperatures.
