# Supplementary material for: Effects of Ball Milling Time and Sintering Temperature on the Microstructure and Mechanical Properties of Mg-Al-Ti Alloy
Source: Materials (Basel). 2025 Oct 29;18(21):4936. doi: 10.3390/ma18214936 (PMC12610678; doi:10.3390/ma18214936)
Supplement: Supplementary file 1 [file materials-18-04936-s001.zip › materials-3892676-supplementary.pdf]

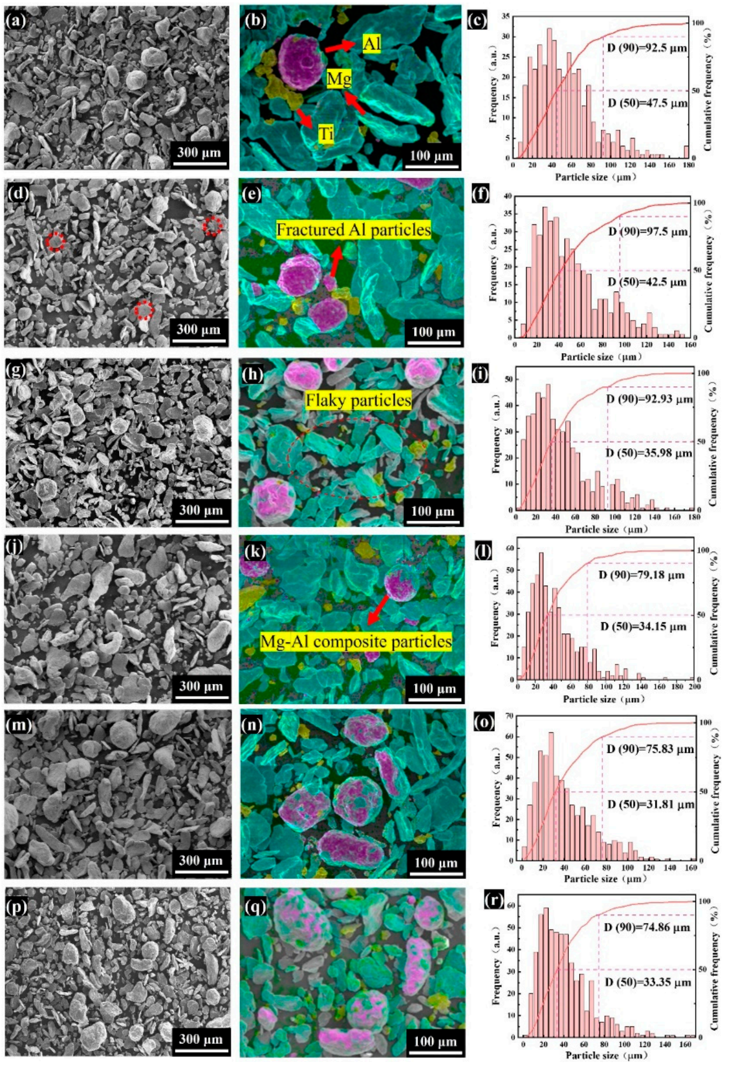

Fig. S1 SEM, EDS and particle size statistics of P1 with different milling time: (a-c) 80 min; (d-f) 240 min; (g-i) 320 min; (j-l) 400 min; (m-o) 480 min; (p-r) 640 min

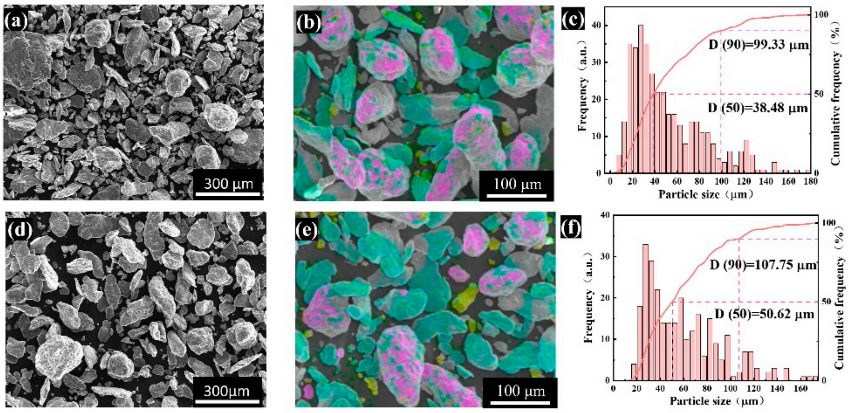

Fig. S2 SEM, EDS and particle size statistics of P2 with different milling time: (a-c) 480min; (d-f) 640 min

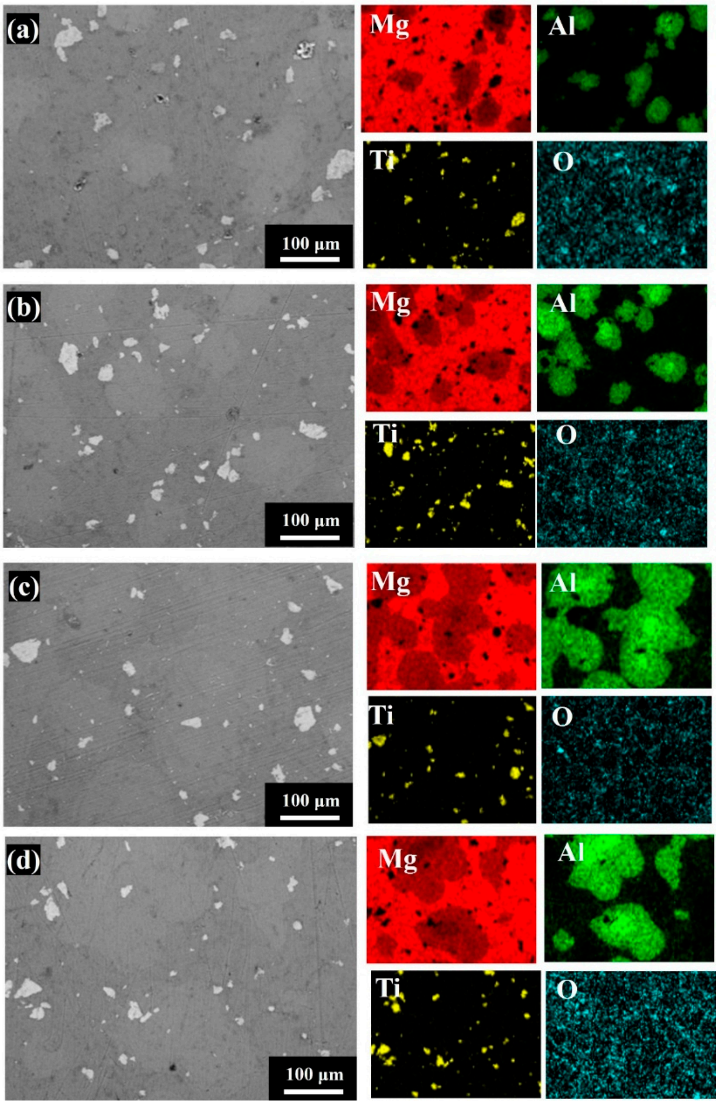

Fig. S3 SEM and EDS results of sintered samples of P1 with different ball milling times: (a) 80 min; (b) 240 min; (c) 480 min; (d) 640 min

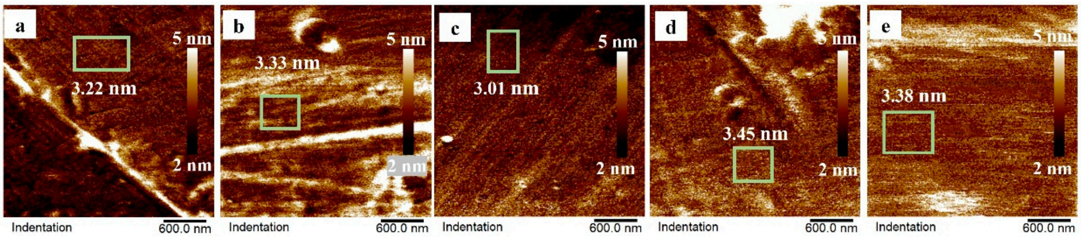

Fig. S4 Indentation maps of P2 at different sintering temperatures: (a) 405 °C; (b) 415 °C; (c) 420 °C; (d) 425 °C; (e) 430 °C

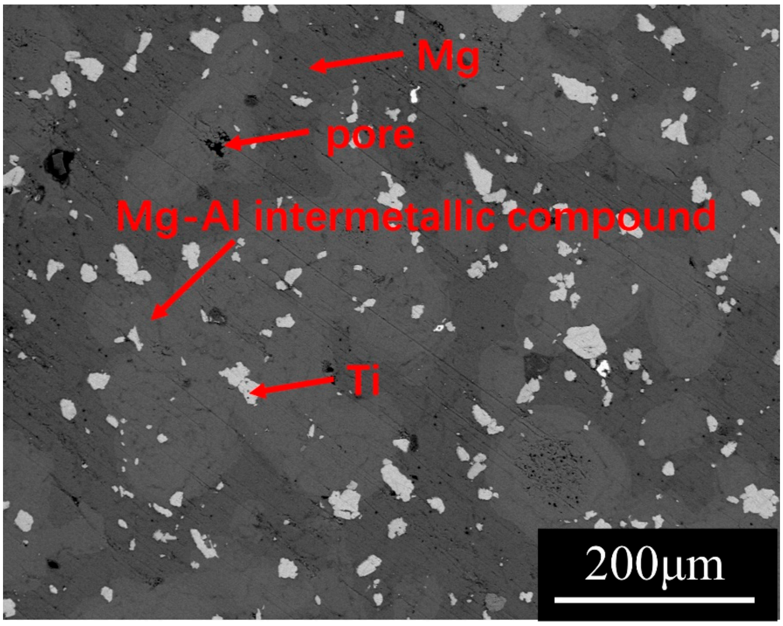

Fig. S5 SEM of the sintered sample at 385 ℃

Table S1 Lattice strain and dislocation density of P1 ball-milled powders

Milling time [min]	FWHM [°]	Lattice strain [nm]	Dislocation density [$10^{15} \cdot m^{-2}$]
80 min	0.1564	1.949	0.427
160 min	0.1542	1.891	0.434
240 min	0.1640	2.047	0.513
320 min	0.1637	2.018	0.499
400 min	0.1640	2.026	0.506
480 min	0.1760	2.203	0.580
560 min	0.1587	1.987	0.475
640 min	0.1587	1.987	0.469

Table S2 Lattice strain and dislocation density of P2 ball-milled powders

Milling time [min]	FWHM [°]	Lattice strain [nm]	Dislocation density [$10^{15} \cdot m^{-2}$]
80 min	0.1486	1.813	0.369
160 min	0.1554	1.911	0.408
240 min	0.1576	1.967	0.429
320 min	0.1755	2.159	0.519
400 min	0.1603	1.989	0.438

480 min	0.1596	1.956	0.424
560 min	0.1574	1.950	0.417
640 min	0.1569	1.942	0.423

Table S3 Theoretical and actual densities of P1 and P2

Milling time [min]	Theoretical density [g·cm^{-3}]		Actual density [g·cm^{-3}]	
	P1	P2	P1	P2
80	1.94	2.18	1.862	1.973
160			1.883	2.005
240			1.909	2.109
320			1.904	2.125
400			1.913	2.082
480			1.922	2.070
560			1.913	2.037
640			1.857	2.024

Table S4 The specific values of compressive strength, compressive elastic modulus, and fracture strain of the P2 alloys sintered at different temperatures

Sintered temperature [°C]	Compressive strength [MPa]	compressive elastic modulus [GPa]	Fracture strain [%]
405	365.44	1.56	32.48
420	382.19	1.51	36.72
430	374.47	1.52	34.35

In the initial phase of the experiment, we conducted exploratory tests within a ball milling time range of 1–9 hours and observed a critical phenomenon: as ball milling time increased, the surface color of sintered samples darkened significantly, accompanied by pronounced uneven layering issues. Complementary particle size analysis confirmed that a 5-hour grinding duration yielded the smallest powder particles and the largest specific surface area. Based on this finding, combined with existing

literature and preliminary experimental conclusions, we adjusted the grinding time range for subsequent precision experiments to 80–640 minutes. A gradient variable was set at 80-minute intervals to more efficiently screen for the optimal process conditions.

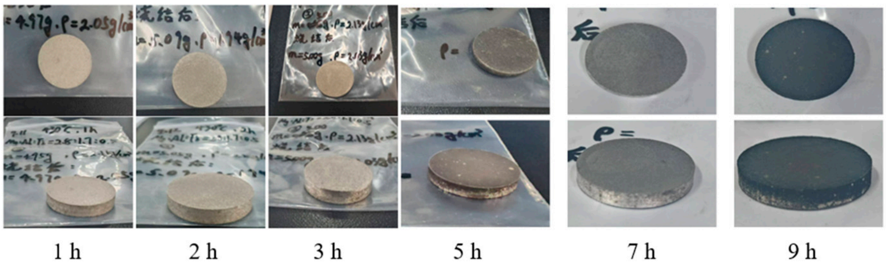

Figure. S6 Macroscopic morphology of samples prepared at different milling times

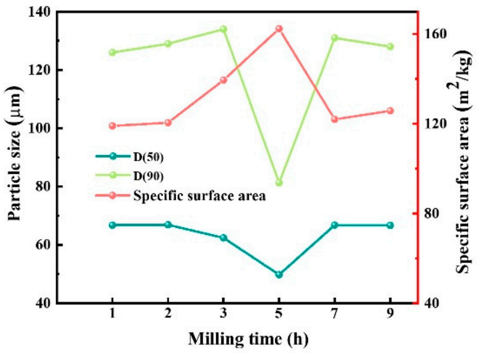

Figure. S7 Particle size of ball-milled powders at varied ball milling times
